# A model for individualized prediction of liver-related death in outpatients with alcohol-associated cirrhosis

**DOI:** 10.1097/HC9.0000000000000229

**Published:** 2023-08-31

**Authors:** Astrid Marot, Jean Henrion, Jean-François Knebel, Eric Trépo, Christophe Moreno, Pierre Deltenre

**Affiliations:** 1 Department of Gastroenterology and Hepatology, CHU UCL Namur, Université Catholique de Louvain, Yvoir, Belgium; 2 Department of Gastroenterology and Hepatology, Hôpital de Jolimont, Haine-Saint-Paul, Belgium; 3 Division of Radiology, Centre d’Imagerie Biomédicale (CIBM), Hôpital Nestlé, Centre Hospitalier Universitaire Vaudois, University of Lausanne, Lausanne, Switzerland; 4 Department of Gastroenterology, Hepatopancreatology and Digestive Oncology, CUB Hôpital Erasme, Université Libre de Bruxelles, Brussels, Belgium; 5 Laboratory of Experimental Gastroenterology, Université Libre de Bruxelles, Brussels, Belgium; 6 Department of Gastroenterology and Hepatology, Clinique St Luc, Bouge, Belgium

## Abstract

**Introduction:**

In alcohol-associated cirrhosis, an accurate estimate of the risk of death is essential for patient care. We developed individualized prediction charts for 5-year liver-related mortality among outpatients with alcohol-associated cirrhosis that take into account the impact of abstinence.

**Methods:**

We collected data on outpatients with alcohol-associated cirrhosis in a prospective registry. The model was derived, internally and externally validated, and compared with the Child-Pugh and the Model For End-Stage Liver Disease (MELD) scores.

**Results:**

A total of 527 and 127 patients were included in the derivation and validation data sets, respectively. A model was developed based on the 3 variables independently associated with liver-related mortality in multivariate analyses (age, Child-Pugh score, and abstinence). In the derivation data set, the model combining age, Child-Pugh score, and abstinence outperformed the Child-Pugh and the MELD scores. In the validation data set, the Brier score was lower for the model (0.166) compared with the Child-Pugh score (0.196, *p* = 0.008) and numerically lower compared with the MELD score (0.190) (*p* = 0.06). The model had the greatest AUC (0.77; 95% CI 0.68–0.85) compared with the Child-Pugh score (AUC = 0.66; 95% CI 0.56–0.76, *p* = 0.01) and was numerically higher than that of the MELD score (AUC = 0.66; 95% CI 0.56–0.78, *p* = 0.06). Also, the Akaike and Bayesian information criterion scores were lower for the model (2163; 2172) compared with the Child-Pugh (2213; 2216) or the MELD score (2205; 2208).

**Conclusion:**

A model combining age, Child-Pugh score, and abstinence accurately predicts liver-related death at 5 years among outpatients with alcohol-associated cirrhosis. In this study, the model outperformed the Child-Pugh and the MELD scores, although the AUC and the Brier score of the model were not statically different from the MELD score in the validation data set.

## INTRODUCTION

Alcohol-related liver disease (ARLD) is a leading cause of chronic liver disease and is associated with significant morbidity and mortality worldwide.^1–5^ An accurate estimate of the prognosis of patients with severe forms of ARLD is essential for patient care. The prognosis of patients with cirrhosis depends on several factors, such as the etiology and severity of liver disease and the presence of complications and comorbidities.^2,3,6–8^ Existing scores allow a rough assessment of the prognosis for chronic liver disease and classify patients into risk categories. The most widely used prognostic scores are the Child-Pugh and the Model For End-Stage Liver Disease (MELD) scores that have similar prognostic values in most patients with cirrhosis in nontransplant settings.^9,10^ However, although they provide some useful information, these scores lack accuracy because the risk categories pool patients at different levels of disease severity together. Currently, prognostic models that provide an individualized estimate of the risk of liver-related death are not available for patients with alcohol-associated cirrhosis.

While it is expected that abstinence from alcohol improves the outcome of patients with alcohol-associated cirrhosis, few studies have been specifically designed to assess the impact of discontinuation of alcohol consumption on prognosis.^11–13^ A meta-analysis including 7 studies observed that abstinence was associated with improved survival if it lasted at least 1.5 years.^14^ However, most of these studies included a limited number of patients followed for a short period of time and were performed many years ago. In addition, outside the specific setting of alcoholic hepatitis, no prognostic models have been developed specifically in alcohol-associated cirrhosis that take into account those who abstained and those who did not abstain from alcohol.

The best model would be one that combines prognostic information from independent variables associated with mortality to provide a continuous prediction of the risk of death. In addition, the model should be easy to use in clinical practice. To develop such a model, a large sample of well-characterized patients is needed to identify independent predictors of death and to evaluate the added value provided by their combined assessment of abstainers and consumers. The overall aim of this study was to develop and validate individualized prediction charts for liver-related mortality at 5 years among outpatients with alcohol-associated cirrhosis that take into account the impact of abstinence. For this purpose, we followed patients with alcohol-associated cirrhosis, consecutively seen in a single center during a 21-year period, and collected data related to alcohol use, death, and causes of death. The secondary aims of the study were to compare the new model to the Child-Pugh and MELD scores and to evaluate overall mortality.

## METHODS

### Study populations

The patients included in this study were grouped into 2 cohorts, a derivation and a validation data set. The derivation cohort included all patients referred from the outpatient clinic of one of the study’s investigators between January 1995 and December 2014 if they fulfilled the following criteria: (1) age > 18 years; (2) cirrhosis demonstrated by liver biopsy showing fibrotic nodules consistent with a METAVIR F4 fibrosis stage or by unequivocal signs of cirrhosis (dysmorphic liver, ascites, esophageal or gastric varices); (3) ARLD. The diagnosis of ARLD was based on the following criteria: (1) history of chronic alcohol intake greater than 210 g/week for men and greater than 140 g/week for women; (2) history of long-standing alcohol consumption; (3) absence of another cause of liver disease. All stages of cirrhosis were included. The validation cohort included all patients consecutively seen between January 2015 and December 2019 in the same center and following the same inclusion criteria. This study was approved by the ethical committee of the center in which the observatory was conducted. Informed consent was waived because this study used anonymous retrospective data.

### Data collection

Baseline data and follow-up data related to alcohol intake, death, and cause of death were recorded. Alcohol consumption was assessed at the time of the first visit (baseline) and at each follow-up visit. The assessment of alcohol consumption was made according to patient declarations and not through the use of a standardized questionnaire.

At baseline, data collected included demographic data (sex, age), clinical data (alcohol consumption and tobacco use, the presence of ascites and encephalopathy, weight, height, and the presence of esophageal varices and diabetes), past medical history, biological data (bilirubin, albumin, creatinine levels, prothrombin time or international normalized ratio, and platelet count), and histological data (presence of fibrotic nodules consistent with a METAVIR F4 fibrosis stage). The Child-Pugh and the MELD score were calculated. The MELD-Na and the MELD 3.0 were also calculated.

During follow-up, patients were followed as outpatients every 6 months, or as frequently as required for the management of chronic liver disease. Data collected included clinical data (current alcohol use) and data related to the development of complications of cirrhosis (HCC), or to the occurrence of liver transplantation or death and cause of death. Deaths due to HCC or decompensation of cirrhosis (either acute-on-chronic liver failure or end-stage liver disease) were considered liver-related. All other causes of death were considered non–liver-related.

Alcohol consumption was coded as present or absent but was not quantified to avoid imprecise data on the amount of alcohol. In case of missing data, patient charts were reviewed, and the patient’s family and/or their general practitioner were contacted. Final data were collected on December 31, 2015 for the derivation data set and on August 31, 2021 for the validation data set.

### Abstinence

Abstinence was defined as the definitive discontinuation of any alcohol intake at inclusion. Hence, patients who did not discontinue alcohol consumption before inclusion or at inclusion were considered to be consumers. This definition was applied for the derivation and the validation data set.

### Statistical analysis

Quantitative variables are expressed as means (SD) if the distribution was normal or medians (95% CI) otherwise. Qualitative variables are expressed as frequencies and percentages. Analyses were conducted using variance analysis, the chi-square test, 2-sided Fisher exact test, Mann-Whitney test, Wilcoxon test, and 2-sample Student *t*-test when appropriate. Follow-up started with the inclusion of patients. For the derivation cohort, data for patients who had not died were censored at the time of last contact or on December 31, 2015, whichever was earlier. For the validation cohort, data for patients who had not died were censored at the time of last contact or on August 31, 2021, whichever was earlier. To avoid having patients misclassified related to their alcohol consumption during the follow-up, a method recently published by Hofer et al^15^ was used in which patients were censored upon changing their baseline alcohol consumption status (ie, chronic relapse in abstinent patients and persistent abstinence in patients with active alcohol intake). Thus, all patients with baseline abstinence subsequently remained abstinent, and all patients with active alcohol intake at baseline demonstrated continued alcohol consumption over the course of the study. Univariate analyses were performed using NCSS 2016 software (NCSS, Kaysville, UT, USA). All other analyses were performed using the *R* statistical language, Vienna, Austria,^16^ Python 3.5 language,^17^ Rpy2 modules, and “cmprsk” R library.^18,19^ Multistate models and cumulative incidence functions were also used, as recommended and described.^3,20^ The risk of death was estimated with the cumulative incidence function taking into account liver transplantation as a competing risk. The risk of liver-related death was estimated with the cumulative incidence function taking into account death from non–liver-related causes and liver transplantation as competing risks.

To develop the best model, we first conducted a univariate and multivariate Fine and Gray proportional hazards model to identify factors associated with overall death and liver-related death. Missing MELD values were imputed using predictive mean matching by Child-Pugh score, sex, and age as recommended and reported.^21–23^ We also performed a sensitivity analysis limited to patients with all available data. To avoid bias related to the effect of colinearity, when Child-Pugh or MELD scores were included in multivariate analysis, their constituent variables were not considered. For the same reason, Child-Pugh and MELD score were not combined in the same multivariate model. Hence, 2 multivariate analyses were performed, 1 including the Child-Pugh score and another including the MELD score. Subdistribution HR are reported with 95% CIs. All tests were 2-tailed and a *p-*value of less than 0.05 was considered to be statistically significant. The combination of the selected variables was used to predict mortality over time according to the following function: 1−exp(−B(t)), where B(t) is the estimated cumulative subdistribution hazard obtained for the specified covariate values obtained from the Breslow-type estimate of the underlying hazard and the estimated regression coefficients. So, variables were extracted to calculate the probability of dying over time to build 2 prediction models combining age, abstinence, and either the Child-Pugh or the MELD score at baseline (see results section for the reason why these variables were chosen). To use the models more easily in clinical practice, charts were designed with contour lines linking the points of equal probability of death grouped by 10% and age grouped in steps of 5 years.

Then, we first performed internal validation by using bootstrap resampling, which consists of drawing random samples with replacement from the derivation cohort, with a sample size equal to that of the original cohort. Here, we used the original data set of 527 patients with their own Child-Pugh and MELD score, age, and abstinence data. The process was repeated 10,000 times. Second, we performed external validation (temporal validation) of the scoring system by assessing model calibration and discrimination performance using the validation data set.

The traditional statistical approach to assess the performance of a prediction model is to quantify how close predictions are to the actual outcome.^24,25^ For that purpose, different measures were used (see Supplemental Material for more details, http://links.lww.com/HC9/A436). All analyses compared the performance of the prediction model combining age, Child-Pugh score, and abstinence to the performance of the different models frequently used in clinical practice (Child-Pugh and MELD scores alone) and to the performance of the prediction model combining age, MELD score, and abstinence to predict liver-related mortality at 5 years. We also compared the model with the MELD-Na and the MELD 3.0.

The Transparent Reporting of a Multivariable Prediction Model for Individual Prognosis or Diagnosis (TRIPOD) checklist for guidance was used. The TRIPOD guidelines provide recommendations for the development and validation of prediction models for diagnosis and prognostic purposes^26^ (Supplemental Material, http://links.lww.com/HC9/A436).

## RESULTS

### Study cohort and outcomes

**FIGURE 1:**
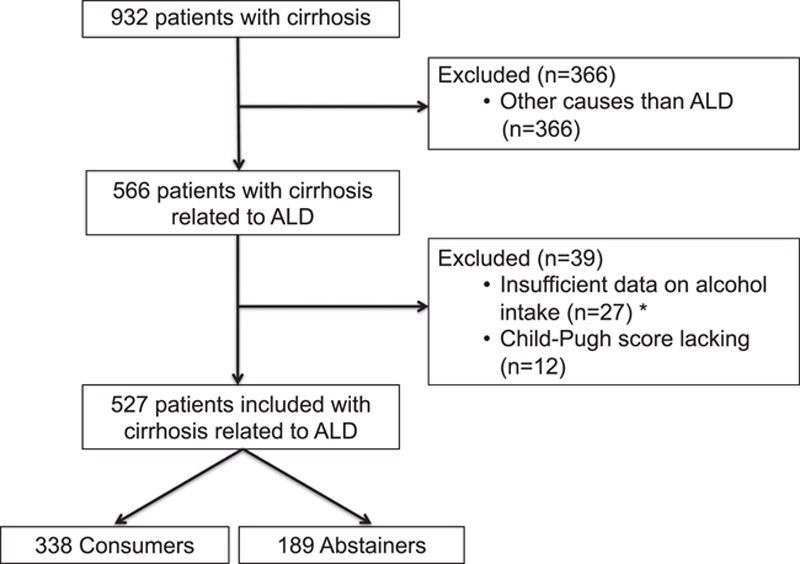
Flowchart of the derivation data set.

From January 1995 to December 2014, 932 patients with cirrhosis were identified, as described.^3^ From January 2015 to December 2019, 214 patients with cirrhosis were identified. Of these patients, 366 patients from the derivation sample and 86 patients from the validation sample were excluded because they had cirrhosis not related to ARLD. The derivation cohort enrolled a total of 566 patients (Figure 1). The validation cohort enrolled a total of 128 patients (Supplemental Figure S1, http://links.lww.com/HC9/A437). Thirty-nine patients from the derivation sample and 1 patient from the validation sample were excluded because of insufficient data on alcohol intake (n = 27 and n = 1, respectively), some of whom were lost to follow-up (n = 10 and n = 0, respectively), or because Child-Pugh score data was lacking (n = 12 and n = 0, respectively). Thus, 527 and 127 patients were included in the derivation data set and in the validation data set, respectively (Figure 1 and Supplemental Figure S1, http://links.lww.com/HC9/A437). The median time from inclusion to the last available data was 50 months (95% CI: 45–57) in the derivation data set, and 46 months (95% CI: 36–50) in the validation data set.

Baseline patient characteristics are shown in Table 1. Among the 527 patients included in the derivation data set, 69% were male, the median age was 56 years (95% CI: 55–57), 47% had Child-Pugh stage B or C disease, and the median MELD score was 9 (95% CI: 9–10). A total of 189 patients had discontinued alcohol consumption at baseline and were considered to be abstainers. The other 338 patients were considered to be consumers. Abstainers were older than consumers (58 vs. 54 y; *p* < 0.001). Otherwise, patient characteristics did not significantly differ between the 2 groups. Among the 127 patients included in the validation data set, a total of 62 patients had discontinued alcohol consumption at baseline and were considered to be abstainers. The other 65 patients were considered to be consumers. Abstainers were older than consumers (62 vs. 59 y; *p* = 0.003). C-reactive protein was higher in consumers than in abstainers (9.2 vs. 2.0 mg/dL; *p* = 0.02). Otherwise, patient characteristics were comparable between the 2 groups (Table 1). Of note, in the derivation data set, 34% of the patients were diabetic and 33% had obesity (body mass index ≥ 30 kg/m^2^). These percentages were quite similar in the validation data set (33% and 38%, respectively). Regarding the interval of abstinence among abstainers, the median time from the date of abstinence to inclusion (ie, the median time from the date of the last drink to the first visit) was similar between the derivation data set and the validation data set (0 days [95% CI: −31 to 0] and 0 days [95% CI: 0–0], respectively). During the follow-up, 8 abstainers (6 in the derivation cohort and 2 in the validation cohort) had a late relapse in alcohol consumption. Similarly, 12 consumers (9 in the derivation cohort, 3 in the validation cohort) were finally able to stop consuming alcohol several months after the inclusion period. According to preplanned analyses, the follow-up of these patients ended when they changed their alcohol behavior.

**TABLE 1:** Baseline characteristics of the study population according to alcohol intake (derivation data set and validation data set)

	Derivation data set				Validation data set			
Characteristics	Overall population (n = 527)	Consumers (n = 338)	Abstainers (n = 189)	*p*	Overall population (n = 127)	Consumers (n = 65)	Abstainers (n = 62)	*p*
Age (y)^a^	56 (55–57)	54 (53–55)	58 (56–60)	< 0.001	60 (58–63)	59 (57–61)	62 (60–66)	0.003
Male sex (no of males, %)	365 (69)	233 (69)	132 (70)	0.8	89 (70)	47 (72)	42 (68)	0.7
Diabetes (no, %)	107 (34)^b^	61 (33)	46 (36)	0.5	42 (33)^j^	21 (32)	21 (34)	1.0
Tobacco use (no of consumers, %)	145 (64)^c^	88 (65)	57 (68)	0.9	50 (56)^k^	26 (58)	24 (53)	0.9
BMI (kg/m²) ^a^	26 (25–28)^d^	27 (25–28)	26 (25–28)	0.7	28 (26–30)^l^	28 (26–31)	28 (26–30)	1.0
Bilirubin levels (mg/dL) ^a^	1.3 (1.2–1.5)	1.2 (1.1–1.5)	1.5 (1.2–1.7)	0.7	1.1 (0.9–1.2)	1.1 (0.9–1.4)	1.0 (0.8–1.2)	0.6
INR^a^	1.1 (1.1–1.1)	1.1 (1.1–1.1)	1.1 (1.1–1.1)	0.9	1.1 (1.1–1.2)	1.1 (1.1–1.2)	1.1 (1.1–1.2)	0.8
Albumin levels (g/dL)^a^	3.7 (3.6–3.9)	3.8 (3.6–3.9)	3.7 (3.4–3.9)	0.4	3.8 (3.5–4.0)	3.9 (3.7–4.0)	3.6 (3.2–3.9)	0.2
Creatinine levels (mg/dL)^a^	0.8 (0.8–0.8)	0.8 (0.8–0.9)	0.8 (0.8–0.9)	1.0	0.8 (0.7–0.9)	0.8 (0.7–0.9)	0.8 (0.7–0.9)	0.7
Platelet count (10^3^/mm³)^a^	142 (133–153)	139 (129–151)	153 (135–169)	0.2	133 (121–154)	133 (119–157)	137 (119–169)	0.3
Ascites (%)	145 (44)^e^	82 (37)	63 (45)	0.2	53 (42)	24/65 (37)	29/62 (47)	0.3
HE (%)	23 (5)^f^	13/273 (5)	10/168 (6)	0.7	10 (8)	3 (5)	7 (11)	0.2
Presence of esophageal or gastric varices (no, %)	198 (58)^g^	122 (58)	76 (58)	1.0	58 (51)^m^	26 (46)	32 (55)	0.5
Child-Pugh score^a^	6 (6–7)	6 (6–7)	6 (6–7)	0.9	6 (5–6)	6 (5–6)	6 (5–7)	0.4
Child-Pugh classification	—	—	—	0.7	—	—	—	0.1
Child-Pugh class A (no, %)	279 (53)	177 (52)	102 (54)	—	80 (63)	43 (66)	37 (60)	—
Child-Pugh class B (no, %)	164 (31)	104 (31)	60 (32)	—	27 (21)	16 (25)	11 (18)	—
Child-Pugh class C (no, %)	84 (16)	57 (17)	27 (14)	—	20 (16)	6 (9)	14 (23)	—
MELD score^a^	9 (9–10)^h^	9 (8–10)	9 (9–10)	0.9	8.0 (8.0–9.0)^n^	8.0 (7–9)	8 (7–10)	0.8
Natremia (mEq/l)	NA	NA	NA	—	138 (137–140)^o^	138 (137–140)	138 (136–140)	0.8
MELD-Na	NA	NA	NA	—	9 (8–11)^p^	8 (8–11)	9 (8–13)	0.6
MELD 3.0	NA	NA	NA	—	11 (9–13))^q^	11 (8–12)	11 (9–15)	0.5
CRP md/dL	1.4 (1.1–1.9)^i^	1.2 (0.8–1.9)	1.8 (1.2–2.9)	0.8	5.1 (2.0–12.3)^r^	9.2 (6.0–24.6)	2.0 (1.7–4.3)	0.02

a Data are expressed as median (95% CI).

b Data available in 314 patients.

c Data available in 219 patients.

d Data available in 176 patients.

e Data available in 331 patients.

f Data available in 441 patients.

g Data available in 340 patients.

h Data available in 348 patients.

i Data available in 187 patients.

j Data available in 125 patients.

k Data available in 90 patients.

l Data available in 92 patients.

m Data available in 114 patients.

n Data available in 127 patients.

o Data available in 99 patients.

p Data available in 99 patients.

q Data available in 98 patients.

r Data available in 94 patients.

Abbreviations: BMI, body mass index; INR, international normalized ratio; MELD, Model For End-Stage Liver Disease; NA, not available.

Data related to death and liver transplantation occurring during the study period in the derivation data set and the validation data set are reported in Supplemental Table S1, http://links.lww.com/HC9/A436. During follow-up, 289 patients included in the derivation data set died (25 from HCC, 170 from liver failure, 90 from non–liver-related causes, 4 from unknown causes) and 19 patients were transplanted, while 54 patients included in the validation data set died (5 from HCC, 33 from liver failure, 13 from non–liver-related causes, 3 from unknown causes) and 4 patients were transplanted. The 5-year cumulative incidence risks of liver-related mortality and overall mortality in abstainers and in consumers included in the derivation data set are reported in Supplemental Figures S2, http://links.lww.com/HC9/A438 and S3, http://links.lww.com/HC9/A439. The 5-year cumulative incidence risks of liver-related mortality were lower in abstainers than in consumers included in the derivation data set (5% vs. 21%, respectively; *p* < 0.001, Supplemental Figure S2, http://links.lww.com/HC9/A438). The 5-year cumulative incidence risks of liver-related mortality were lower in abstainers than in consumers included in the validation data set (8% vs. 21%, respectively; *p* = 0.002).

### Prediction models for liver-related death

#### Risk prediction charts

According to preplanned analyses, 2 models were derived based on the derivation cohort using multivariate Fine and Gray proportional hazards models (see statistical analyses section for more details). The first model included the Child-Pugh score and the second included the MELD score.

##### First model including the Child-Pugh score

**FIGURE 2:**
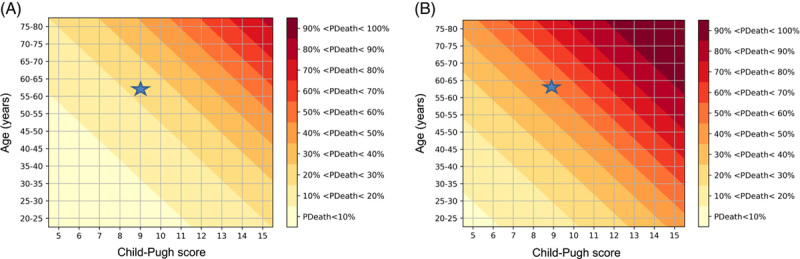
Chart predicting the risk of liver-related death at 5 years in patients who abstained from alcohol (A) and who did not abstain from alcohol (B). Legend:☆, hypothetical 60-year-old patient with a Child-Pugh score of 9 at baseline who abstained from alcohol: 5-year liver-related mortality rate is 23%. Legend:☆, hypothetical 60-year-old patient with a Child-Pugh score of 9 at baseline who did not abstain from alcohol: 5-year liver-related mortality rate is 51%.

In this multivariate model, 3 variables available at baseline and associated with liver-related death were used to calculate the probability over time: age, alcohol behavior, and Child-Pugh score (Table 2). To use the prediction model more easily in clinical practice, charts were designed with contour lines linking the points of equal probability of death grouped by 10% and age grouped by steps of 5 years. Figure 2 shows how the model converts any individual score (age and Child-Pugh score) into a risk of liver mortality at 5 years according to the value of the other variable among abstainers (Figure 2A) and consumers (Figure 2B). For any combination of age and Child-Pugh score, patients who did not abstain from alcohol had an ~2-fold higher risk of dying at 5 years than patients who abstained from alcohol. The example of a 60-year-old patient with a Child-Pugh score at 9 at baseline is plotted in Figure 2A and B.

**TABLE 2:** Risk factors for death

		Overall mortality				Liver-related mortality			
		Univariate		Multivariate		Univariate		Multivariate	
Baseline characteristics	Comparison group	SHR (95% CI)	*p*	SHR (95% CI)	*p*	SHR (95% CI)	*p*	SHR (95% CI)	*p*
Age	1-year increase	1.03 (1.02–1.05)	< 0.001	1.04 (1.03–1.06)	< 0.001	1.02 (1.00–1.03)	0.001	1.04 (1.02–1.05)	< 0.001
Sex	Male vs. female	1.40 (1.09–1.78)	0.008	1.30 (0.91–1.85)	0.2	1.23 (0.93–1.63)	0.14	—	—
Diabetes	Yes vs. no	1.07 (0.76–1.51)	0.7	—	—	0.98 (0.67–1.45)	0.9	—	—
Tobacco use	Yes vs. no	0.92 (0.60–1.41)	0.7	—	—	0.83 (0.51–1.36)	0.5	—	—
BMI	1 kg/m^2^ increase	0.99 (0.95–1.04)	0.7	—	—	1.03 (0.98–1.09)	0.2	—	—
Bilirubin	1 mg/dL increase	1.09 (1.02–1.17)	0.008	—	—	1.11 (1.04–1.19)	0.003	—	—
INR	1-point increase	4.46 (2.27–8.77)	< 0.001	—	—	6.95 (3.39–14.26)	< 0.001	—	—
Albumin	1 g/dL increase	0.90 (0.88–0.93)	< 0.001	—	—	0.90 (0.87–0.93)	< 0.001	—	—
Creatinine	1 mg/dL increase	4.70 (2.82–7.84)	< 0.001	—	—	4.65 (2.65–8.13)	< 0.001	—	—
Platelet count	10^3^/mm³ increase	1.00 (1.00–1.00)	0.7	—	—	1.00 (1.00–1.00)	0.7	—	—
Ascites	Yes vs. no	1.60 (1.18–2.17)	0.003	—	—	1.99 (1.41–2.82)	< 0.001	—	—
Encephalopathy	Yes vs. no	2.15 (1.11–4.18)	0.02	—	—	2.64 (1.40–4.99)	0.003	—	—
Esophageal or gastric varices	Yes vs. no	1.39 (0.99–1.94)	0.05	—	—	1.49 (1.00–2.19)	0.05	—	—
Child-Pugh score	1-point increase	1.12 (1.06–1.18)	< 0.001	1.13 (1.07–1.20)	< 0.001	1.19 (1.13–1.26)	< 0.001	1.21 (1.14–1.29)	< 0.001
MELD score	1-point increase	1.10 (1.05–1.15)	< 0.001	—	—	1.13 (1.07–1.18)	< 0.001	—	—
Abstinence	Yes vs. no	0.48 (0.37–0.62)	< 0.001	0.44 (0.33–0.57)	< 0.001	0.41 (0.29–0.57)	< 0.001	0.37 (0.26–0.53)	< 0.001

*Note:* In time-dependent multivariate proportional hazards models, factors associated with liver-related mortality were older age (SHR: 1.04; 95% CI, 1.02–1.05; *p* < 0.001), abstinence (SHR: 0.37; 95% CI, 0.26–0.53; *p* < 0.001), and Child-Pugh score (SHR: 1.21; 95% CI, 1.14–1.29; *p* < 0.001). Factors associated with overall mortality were older age (SHR: 1.04; 95% CI, 1.03–1.06; *p* < 0.001), abstinence (SHR: 0.44; 95% CI, 0.33–0.57; *p* < 0.001), and baseline Child-Pugh score (SHR: 1.13; 95% CI, 1.07–1.20; *p* = *p* < 0.001).

Abbreviations: BMI, body mass index; INR, international normalized ratio; MELD, Model For End-Stage Liver Disease; SHR, subdistribution HR.

##### Second model including the MELD score

In this multivariate model, 3 variables available at baseline and associated with liver-related death were used to calculate the probability over time: age, alcohol behavior, and MELD score (Supplemental Table S2, http://links.lww.com/HC9/A436). Charts were designed with contour lines linking the points of equal probability of death grouped by 10% and age grouped by steps of 5 years. Supplemental Figure S4, http://links.lww.com/HC9/A440, http://links.lww.com/HC9/A441 shows how the model converts any individual score (age and MELD score) into a risk of liver mortality at 5 years according to the value of the other variable among abstainers (Supplemental Figure S4A, http://links.lww.com/HC9/A440) and consumers (Supplemental Figure S4B, http://links.lww.com/HC9/A441). For any combination of age and MELD score, patients who did not abstain from alcohol had a higher risk of dying at 5 years than patients who abstained from alcohol.

#### Validation and performance of the prediction models

In the derivation data set, the Brier score was lower for the model combining age, Child-Pugh score, and abstinence (0.161) compared with the Brier score for the Child-Pugh score alone (0.176, *p* = 0.01) or the MELD score alone (0.175, *p* = 0.02) and similar to the Brier score for the model combining age, MELD score, and abstinence (0.158, *p* = 0.7) (Table 3). In the validation data set, the Brier score was also lower for the model combining age, Child-Pugh score, and abstinence (0.166) compared with the Brier score for the Child-Pugh score alone (0.196, *p* = 0.008) and similar to the Brier score for the model combining age, MELD score, and abstinence (0.162, *p* = 0.6). The Brier score was lower for the model combining age, Child-Pugh score, and abstinence compared with the Brier score for the MELD score alone (0.190), but the difference did not reach significance (*p* = 0.06) (Table 3). Of note, the Brier score was lower for the model combining age, Child-Pugh score, and abstinence compared with the Brier score for the MELD-Na alone (0.206, *p* = 0.03) or the MELD score alone (0.209, *p* = 0.02). The performance of the different prognostic models for 5-year liver-related death for the validation cohort comparing the model to the MELD-Na or the MELD 3.0 is provided in Supplemental Table S3, http://links.lww.com/HC9/A436.

**TABLE 3:** Performance of the different prognostic models for 5-year liver-related death

	Derivation data set						Validation data set			
Models	AUC (95% CI)	*p*	Brier score	*p*	AIC	BIC	AUC (95% CI)	*p*	Brier score	*p*
Age, Child-Pugh score, and abstinence at baseline	0.72 (0.67–0.78)	—	0.161	—	2163	2172	0.77 (0.68–0.85)	—	0.166	—
Child-Pugh score at baseline	0.64 (0.59–0.70)	< 0.001	0.176	0.01	2213	2216	0.66 (0.56–0.76)	0.01	0.196	0.01
MELD score at baseline	0.67 (0.61–0.72)	0.03	0.175	0.02	2205	2208	0.67 (0.56–0.78)	0.06	0.190	0.06
Age, MELD score, and abstinence at baseline	0.74 (0.69–0.79)	0.08	0.158	0.7	2152	2160	0.75 (0.66–0.84)	0.5	0.162	0.6

*Note:* Brier, AIC, and BIC scores provide a global evaluation of the model: a lower Brier score, a lower AIC score, and a lower BIC score indicate better performance.

Abbreviations: AIC, Akaike’s information criterion; BIC, Bayesian information criterion; MELD, Model For End-Stage Liver Disease.

In the derivation data set, a comparison of the Akaike information criterion (AIC) and Bayesian information criterion (BIC) values of the different models calculated from the same sample showed that the model including age, Child-Pugh score, and abstinence had lower AIC and BIC (AIC, 2163; BIC, 2172) than that using the Child-Pugh score alone (AIC, 2213; BIC, 2216) or the one using the MELD score alone (AIC, 2205; BIC, 2208), showing that the former was the best-fitting prognostic model. AIC and BIC values of the model, including age, MELD score, and abstinence, were slightly lower (AIC, 2152; BIC, 2160) than those for the model combining age, Child-Pugh score, and abstinence (Table 3).

**FIGURE 3:**
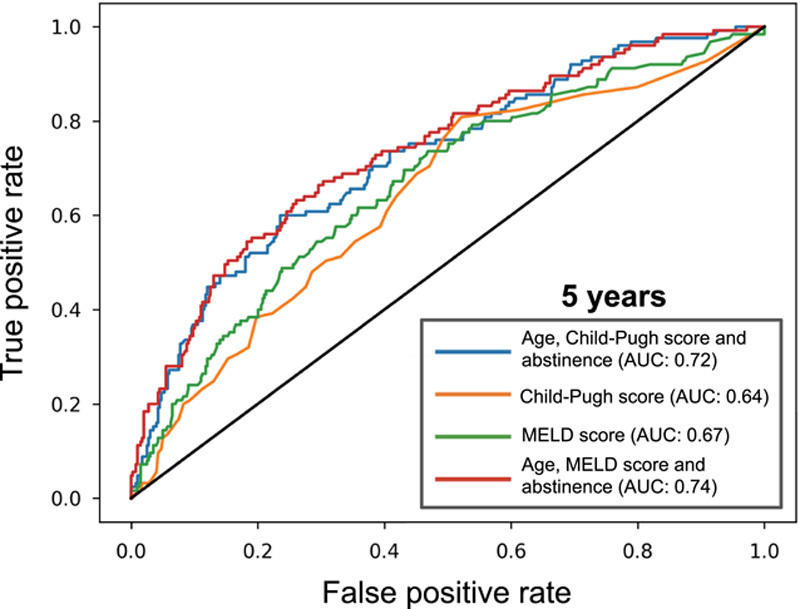
Receiver operating characteristic curves of the different models in the derivation data set.

Regarding discrimination, the model combining age, Child-Pugh score, and abstinence had better AUC (0.72; 95% CI 0.67–0.78) compared with the Child-Pugh score alone (AUC = 0.64; 95% CI 0.59–0.70, *p* < 0.001) or the MELD score alone (AUC = 0.67; 95% CI 0.61–0.72, *p* = 0.03) in the derivation data set (Table 3). The AUC of the model, including age, Child-Pugh score, and abstinence, was similar to the one using the model combining age, MELD score, and abstinence (AUC = 0.74; 95% CI 0.69–0.79, *p* = 0.08). The receiver operating characteristic curves showing the performance of the different models in the derivation data set are shown in Figure 3. In the validation data set, the model combining age, Child-Pugh score, and abstinence had the greatest AUC (0.77; 95% CI 0.68–0.85) compared with the Child-Pugh score alone (AUC = 0.66; 95% CI 0.56–0.76, *p* = 0.01). The model combining age, Child-Pugh score, and abstinence also had the greatest AUC compared with the MELD score, but the difference did not reach significance (AUC = 0.67; 95% CI 0.56–0.78, *p* = 0.06). The AUC of the model, including age, Child-Pugh score, and abstinence, was similar to that of the model combining age, MELD score, and abstinence (AUC = 0.75; 95% CI 0.66–0.84, *p* = 0.5). The receiver operating characteristic curves showing the performance of the different models in the validation cohort are shown in Supplemental Figure S5, http://links.lww.com/HC9/A442. Of note, the model combining age, Child-Pugh score, and abstinence had the greatest AUC compared with the MELD-Na (AUC = 0.62; 95% CI 0.50–0.74, *p* = 0.05) or the MELD 3.0 alone (AUC = 0.63; 95% CI 0.51–0.75, *p* = 0.06) but the difference did not reach significance (Supplemental Table S3, http://links.lww.com/HC9/A436).

The comparison of the model includes the MELD-Na or the MELD 3.0. instead of the MELD score. The model includes the MELD-Na or the MELD 3.0. instead of the MELD score into the model performed as well as the model combining age, Child-Pugh score, and abstinence.

**FIGURE 4:**
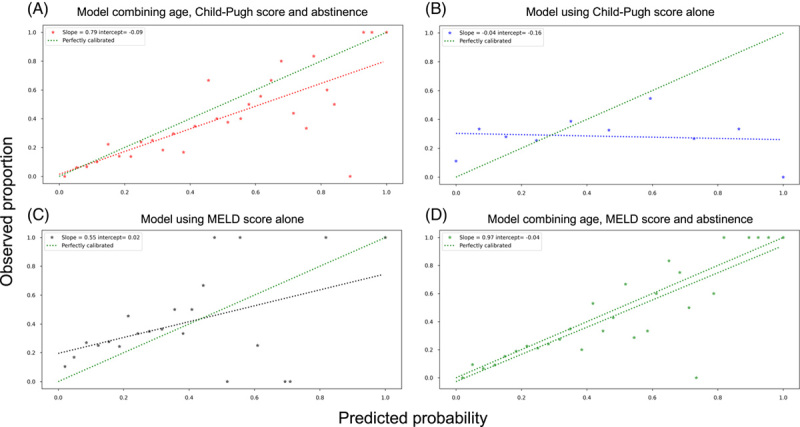
Calibration plots for the models combining (A) age, Child-Pugh score, and abstinence, (B) MELD score alone, (C) Child-Pugh score alone, and (D) age, MELD score, Child-Pugh score, and abstinence in the derivation data set. The calibration slope evaluates the spread of the estimated risks and has a target value of 1. A slope < 1 suggests that estimated risks are too extreme, that is, too high for patients who are at high risk (‘overfitting’). A slope > 1 suggests the opposite (‘underfitting’). The calibration intercept has a target value of 0, negative values suggest overestimation, whereas positive values suggest underestimation. Abbreviation: MELD, Model For End-Stage Liver Disease

Calibration in the derivation data set is shown in Figure 4. For the model combining age, Child-Pugh score, and abstinence (Figure 4A), the curve was better calibrated than that of the model using the Child-Pugh score alone (Figure 4B) or MELD score alone (Figure 4C). Calibration was similar for the model combining age, Child-Pugh score, and abstinence, and the model using age, MELD score, and abstinence (Figure 4D). The model combining age, Child-Pugh score, and abstinence suggested no overfitting (slope 0.79) but slightly overestimated risk predictions for liver-related mortality (intercept – 0.09). The results of the calibration curves in the validation data set are provided in Supplemental Figure S6, http://links.lww.com/HC9/A443.

The accuracy of the model combining age, Child-Pugh score, and abstinence was not significantly affected by a sensitivity analysis limited to patients with all available data (Supplemental Table S4, http://links.lww.com/HC9/A436). The absolute values of the Brier score, AIC, BIC, and receiver operating characteristic were similar to those observed when missing data were imputed. *p*-values for the comparison between the model, including age, Child-Pugh score, and abstinence, and other models identified similar significant differences or at least comparable trends.

### Prediction models for overall death

Similar prediction models for overall death were developed (Table 2). The model combining age, Child-Pugh score, and abstinence was independently associated with the risk of death. Results of all analyses assessing the performance of the models tested are presented in Supplemental Table S5, http://links.lww.com/HC9/A436. Overall, the performance of the model combining Child-Pugh score, age, and abstinence was better than that for Child-Pugh or MELD score alone and similar to that of the model combining age, MELD score, and abstinence.

## DISCUSSION

This study is the first to develop and validate a model that predicts 5-year liver-related mortality in patients with alcohol-associated cirrhosis in an outpatient setting. We provide charts showing the individual risk of liver-related mortality according to age, Child-Pugh score, and the patient’s alcohol behavior, three readily available variables that can be set when a patient is first seen. Overall, the model combining age, Child-Pugh score, and abstinence was clinically useful and provided the best prognostic accuracy and the best discriminative ability for evaluating 5-year liver-related mortality compared with the Child-Pugh score or the MELD score alone.

The demonstration that individual prediction of the risk of dying in the short term helps the management of patients with severe alcoholic hepatitis has highlighted the importance of having similarly accurate prognostic tools in alcohol-associated cirrhosis.^27^ Individual assessment of the risk of death provides prognostic information that is more helpful for clinicians than a rough assessment based on predictive scores that classify patients only into risk categories. Previous studies in the field of alcohol-associated cirrhosis have identified variables associated with mortality, assessed the impact of abstinence on prognosis, or attempted to evaluate the prognosis of patients.^2,13,28,29^ However, with the exception of those focusing on patients with alcoholic hepatitis,^27^ none have provided an individualized assessment of the risk of death.

The model combining age, Child-Pugh score, and abstinence at baseline outperformed the Child-Pugh and MELD scores, the 2 scores widely used in clinical practice for prognostic assessment among patients with cirrhosis.^9^ We acknowledge that the difference between the AUC of the model combining age, Child-Pugh score, and abstinence was not statistically different than that of the MELD score alone in the validation data set. This lack of significance is likely related to a type II error, as the validation cohort included a limited number of patients. We also recognize that the imputation of data for missing MELD scores might have introduced some bias. However, the accuracy of the model was not affected by a sensitivity analysis limited to patients with all available data. Moreover, the accuracy of the model was maintained in the validation cohort, where the MELD score was available for all of the patients. It should be emphasized that the performance of a predictive model does not rely only on AUC values but also on other tests such as those used in this study (ie, Brier score, AIC, and BIC). Hence, even if the AUC of the model, including age, Child-Pugh score, and abstinence, was not significantly different for the prediction of liver-related death from that of the MELD score in the validation cohort, a lot of data support the usefulness and better performance of the new model over the Child-Pugh score or the MELD score. Of note, similar findings were observed for the prediction of overall death, which makes us confident in the study results. In the end, these results emphasize that many factors other than those included in the MELD score or the Child-Pugh score impact mortality. Other studies have already tried to improve upon these scores for predicting outcomes of patients with complications of cirrhosis.^30–32^ Here, we have provided a model that predicts the 5-year liver-related mortality of patients with alcohol-associated cirrhosis better than the Child-Pugh score or the MELD score and can easily be calculated using readily available variables. Moreover, including the MELD score instead of the Child-Pugh score in the model provides a similar predictive value. Importantly, our study emphasizes the huge impact of abstinence in these patients. As an important role of prediction models is to inform patients about their prognosis, we believe that this study could help in insuring the abstinence of patients with alcohol-associated cirrhosis.

We acknowledge that this study has some limitations, particularly because of its retrospective nature, which is why some data were missing. Nevertheless, the primary data were prospectively collected. However, we acknowledge that our results cannot be extrapolated to populations of hospitalized patients with other characteristics. The monocentric design of the study is also a limitation. In addition, therapeutic interventions have changed significantly during the study period, and we were unable to assess their impact on patient outcomes. Furthermore, the results of this study may have been influenced by changes in evolving clinical practice over the long inclusion period. Also, questionnaires were not used to record alcohol consumption. Therefore, underreporting of alcohol consumption and recall bias are possible. Defining abstinence as a binary variable may represent a source of heterogeneity, as a patient who stays abstinent over a long period of time will not have the same benefit from alcohol abstinence as a patient who remains abstinent for a short period of time. However, as most of the patients became abstinent at inclusion, such a bias is unlikely. Finally, neither the body mass index nor diabetes was associated with overall mortality or liver-related mortality in univariate analyses, a finding that may be related to the limited number of patients with available data. We acknowledge that the components of the metabolic syndrome (ie, obesity and diabetes) frequently coexist in patients with alcohol-associated cirrhosis (as in more than 30% of patients in our study) and may influence outcomes, as there is a lot of evidence demonstrating that alcohol consumption interacts with components of the metabolic syndrome to exert a synergic effect on the development and progression of liver disease.^33–36^ Similarly, smoking is generally considered as a significant risk factor for the progression of ARLD.^37^ In our study, tobacco use was also not associated with either overall or liver-related mortality in univariate analyses, but once again, it is possible that this might be the result of a lack of statistical power. Conversely, our study has several robust strengths. Since patients were included in a single center by a sole investigator, it could be expected that the inclusion process was exhaustive, homogeneous, and rigorous according to well-defined criteria. In addition to the long follow-up period and the high number of patients included in the derivation data set, only 10 patients were lost to follow-up and were excluded from the analysis. As a result, a detailed analysis of the causes of death was performed, enabling us to study all causes of mortality as a single outcome as well as liver-related mortality using cumulative incidence functions that took into account competing risks, as recommended.^20^ Finally, we used internal and external validation, as it is recommended when a predictive model is built.^24,38^

In summary, we developed and validated a model combining age, Child-Pugh score, and abstinence which accurately predicts the individual risk of liver-related death at 5 years among patients with alcohol-associated cirrhosis in an outpatient setting. In this study, the prediction model combining age, Child-Pugh score, and abstinence at baseline outperformed the Child-Pugh and the MELD scores, although the AUC and the Brier score of the model were not statically different from the MELD score in the validation data set. A model combining age, MELD score, and abstinence displayed similar predictive performance. An important role of prediction models is to inform patients about their prognosis. These models may serve as a tool for individualized prognostic assessment in daily practice and may help clinicians to motivate patients to stop drinking.

## AUTHOR CONTRIBUTIONS

Astrid Marot: study concept and design; acquisition, analysis, and interpretation of data; and drafting and critical revision of the manuscript for important intellectual content. Jean Henrion: acquisition, analysis, and interpretation of data and critical revision of the manuscript for important intellectual content. Jean-François Knebel: statistical analysis; analysis and interpretation of data; and critical revision of the manuscript for important intellectual content. Eric Trepo and Christophe Moreno: critical revision of the manuscript for important intellectual content. Pierre Deltenre: study concept and design; statistical analysis; analysis and interpretation of data; drafting of the manuscript; critical revision of the manuscript for important intellectual content; and study supervision. All authors approved the final version of the manuscript.

### ACKNOWLEDGMENTS

The authors thank the medical writer, Sandy Field, PhD, for her assistance concerning English-language editing.

### CONFLICTS OF INTEREST

Christophe Moreno consults and received grants from Gilead. He consults for Astellas, Bayer, Echosens, Intercept, Novartis, and Surrozen. He received grants from AbbVie. The remaining authors have no conflicts to report.

## Supplementary Material
