# Supplementary material for: A model for individualized prediction of liver-related death in outpatients with alcohol-associated cirrhosis
Source: Hepatol Commun. 2023 Aug 31;7(9):e0229. doi: 10.1097/HC9.0000000000000229 (PMC10476762; doi:10.1097/HC9.0000000000000229)
Supplement: Supplementary file 1 [file hc9-7-e0229-s001.docx]

**Supplementary Material**

A model for individualized prediction of liver-related death in outpatients with alcohol-associated cirrhosis

Astrid Marot^1^, Jean Henrion^2^, Jean-François Knebel^3^, Eric Trépo^4,5^, Christophe Moreno^4,5^, Pierre Deltenre^1,4,6^

^1^ Department of Gastroenterology and Hepatology, CHU UCL Namur, Université Catholique de Louvain, Yvoir, Belgium

^2^ Department of Gastroenterology and Hepatology, Hôpital de Jolimont, Haine-Saint-Paul, Belgium

^3^ Division of Radiology, Centre d’Imagerie Biomédicale (CIBM), Hôpital Nestlé, Centre Hospitalier Universitaire Vaudois, University of Lausanne, Lausanne, Switzerland

^4^ Department of Gastroenterology, Hepatopancreatology and Digestive Oncology, CUB Hôpital Erasme, Université Libre de Bruxelles, Brussels, Belgium

^5^ Laboratory of Experimental Gastroenterology, Université Libre de Bruxelles, Brussels, Belgium

^6^ Department of Gastroenterology and Hepatology, Clinique St Luc, Bouge, Belgium

**Table of contents**

- **Statistical analyses: assessment of the performance of a prediction model**
- Supplementary Table 1. Clinical events (deaths, hepatocellular carcinoma and liver transplantation) according to alcohol intake during follow-up
- Supplementary Table 2. Risk Factors for death (model including the MELD score in the multivariate analyses)
- **Supplementary Table 3. Performance of the different prognostic models for 5-year liver-related death for the validation cohort comparing the MELD-Na or the MELD 3.0**
- Supplementary Table 4. Performance of the different prognostic models for 5-year liver-related death with all available data
- Supplementary Table 5. Performance of the different prognostic models for 5-year overall death
- Supplementary Figure 1. Flowchart of the validation dataset
- Supplementary Figure 2. Cumulative incidence risk of liver-related mortality among abstainers and consumers during follow-up in the derivation dataset
- Supplementary Figure 3. Cumulative incidence risk of overall mortality among abstainers and consumers during follow-up in the derivation dataset
- Supplementary Figure 4. Charts predicting the risk of liver-related death at 5 years in patients who abstained from alcohol (Supplementary Figure 4A) and in patients who did not abstain from alcohol (Supplementary Figure 4B) with the model including the MELD score instead of the Child-Pugh score.
- Supplementary Figure 5. The ROC curves of the different models in the validation dataset.
- Supplementary Figure 6. Calibration curves in the validation dataset. Calibration plots for the models combining (A) age, Child-Pugh score, and abstinence, (B) Child-Pugh score alone, (C) MELD score alone, and (D) age, MELD score, Child-Pugh score, and abstinence.
- TRIPOD guidelines: The TRIPOD guidelines provide recommendations for development and validation of prediction models for diagnosis and prognostic purposes.

**Statistical analyses: assessment of the performance of a prediction model**

**The traditional statistical approach to assess the performance of a prediction model is to quantify how close predictions are to the actual outcome (1, 2). For that purpose, different measures were used. We used the Brier score to evaluate the overall model performance (3), the area under the receiver operating characteristic (ROC) curve (AUC) to evaluate the discriminative ability, and the “goodness-of-fit” statistic to evaluate calibration (2). The Brier score is a function that measures the accuracy of probabilistic predictions, the model with the lowest Brier score providing the best prediction (2, 4). Of note, the Brier score simultaneously captures discrimination and calibration (2) and is regularly assessed in clinical prediction models (5-7). Comparisons between the Brier score were done using a nonparametric procedure (8). Discrimination refers to the ability to rank patients according to their risk of developing the outcome, whereas calibration refers to the agreement between observed outcomes and predictions (9).** **The discriminative ability was assessed by using the AUC, indicating to what extent the model distinguishes patients who will die from those who will survive. Predictive accuracy was assessed by comparison of AUC curves using the z test comparing the AUC to 0.5, since the AUC of a ‘useless’ criterion is 0.5. Comparisons between the AUCs were done using the Delong method (10, 11). ROC curves showing the performance of the different models are also displayed. Calibration findings were assessed graphically with calibration plots. These plots show the observed proportion of events associated with a model’s predicted risk (12, 13). We focused on the calibration slope as a part of the assessment of statistical overfitting (2, 12). The calibration plot was characterized by an intercept, a, which indicates the extent that predictions are systematically too low or too high, and a calibration slope, b, which should be 1. We also used Akaike Information Criterion (AIC) and Bayesian Information Criterion (BIC) values to compare the performance of the different models: a lower BIC and a lower AIC score indicate better model fit (14, 15).**

**Supplementary Table 1: Clinical events (deaths, hepatocellular carcinoma and liver transplantation) according to alcohol intake during follow-up**

|  | **Derivation dataset** | | | | **Validation dataset** | | | |
| --- | --- | --- | --- | --- | --- | --- | --- | --- |
| **Characteristics** | **Overall population**  **(n=527)** | **Consumers**  **(n=338)** | **Abstainers**  **(n=189)** | ***p-Value*** | **Overall population**  **(n=127)** | **Consumers**  **(n=65)** | **Abstainers**  **(n=62)** | ***p-Value*** |
| Deaths (no., %) | 289 (55%) | 217 (64%) | 72 (38%) | <0.001 | 54 (43%) | 36 (55%) | 18 (33%) | 0.004 |
| Liver-related deaths (no., % of deaths) | 195 (67%) | 156 (72%) | 39 (61%) | 0.05 | 38 (70%) | 27 (75%) | 11 (61%) | 0.02 |
| HCC-related deaths (no., % of deaths) | 25 (9%) | 16 (7%) | 9 (13%) |  | 5 (9%) | 1 (3%) | 4 (29%) |  |
| Non-HCC liver-related deaths (no., % of deaths) | 170 (59%) | 140 (65%) | 30 (49%) |  | 33 (61%) | 26 (72%) | 7 (39%) |  |
| End-stage liver disease (no., % of deaths) | 72 (25%) | 53 (24%) | 19 (26%) |  | 13 (24%) | 10 (28%) | 3 (16%) |  |
| Acute-on-chronic liver failure (no., % of deaths) | 98 (34%) | 87 (40%) | 11 (15%) |  | 20 (37%) | 16 (44%) | 4 (29%) |  |
| Gastrointestinal bleeding (no., % of deaths) | 34 (12%) | 30 (14%) | 4 (6%) |  | 3 (6%) | 2 (6%) | 1 (6%) |  |
| Alcoholic hepatitis (no., % of deaths) | 28 (10%) | 28 (13%) | 0 |  | 8 (15%) | 8 (22%) | 0 |  |
| Sepsis (no., % of deaths) | 20 (7%) | 17 (8%) | 3 (4%) |  | 6 (11%) | 4 (11%) | 2 (11%) |  |
| Others (no., % of deaths) | 16 (6%) | 12 (6%) | 4 (6%) |  | 3 (6%) | 2 (6%) | 1 (6%) |  |
| Non-liver-related deaths (no., % of deaths) | 90 (31%) | 58 (27%) | 32 (44%) |  | 13 (24%) | 9 (25%) | 4 (22%) |  |
| Extra-hepatic malignancies | 31 (11%) | 23 (10%) | 8 (11%) |  | 3 (6%) | 2 (6%) | 1 (6%) |  |
| Strokes | 3 (1%) | 2 (1%) | 1 (1%) |  | 2 (4%) | 2 (6%) | 0 |  |
| Cardio-vascular diseases | 16 (6%) | 5 (2%) | 11 (14%) |  | 2 (4%) | 1 (3%) | 1 (6%) |  |
| Advanced pulmonary diseases | 4 (1%) | 3 (1%) | 1 (1%) |  | 1 (2%) | 0 | 1 (6%) |  |
| Accidental death | 17 (6%) | 12 (6%) | 5 (7%) |  | 3 (6%) | 2 (6%) | 1 (6%) |  |
| Other causes | 19 (7%) | 13 (6%) | 6 (8%) |  | 2 (4%) | 2 (6%) | 0 |  |
| Death from unknown cause (no., % of deaths) | 4 (2%) | 3 (1%) | 1 (2%) |  | 3 (6%) | 1 (3%) | 2 (11%) |  |
| HCC (no., %) | 63 (12%) | 39 (12%) | 24 (13%) | 0.7 |  | 5 (8%) | 10 (16%) | 0.2 |
| Liver transplantation (no., %) | 19 (4%) | 6 (2%) | 13 (7%) | 0.006 | 4 (3%) | 1 (2%) | 3 (5%) | 0.4 |

Abbreviations: CI, confidence interval; HCC, hepatocellular carcinoma

**Supplementary Table 2: Risk Factors for death (model including the MELD score in the multivariate analyses)**

|  |  | **Overall mortality** | | | | **Liver-related mortality** | | | |
| --- | --- | --- | --- | --- | --- | --- | --- | --- | --- |
|  |  | **Univariate** | | **Multivariate** | | **Univariate** | | **Multivariate** | |
| **Baseline characteristics** | **Comparison group** | **Subdistribution hazard ratio**  **(95% CI)** | ***p-Value*** | **Subdistribution hazard ratio**  **(95% CI)** | ***p-Value*** | **Subdistribution hazard ratio**  **(95% CI)** | ***p-Value*** | **Subdistribution hazard ratio**  **(95% CI)** | ***p-Value*** |
| Age | 1-year increase | 1.03 (1.02 – 1.05) | <0.001 | 1.04 (1.03-1.05) | <0.001 | 1.02 (1.00 – 1.03) | 0.001 | 1.04 (1.02 –1.05) | <0.001 |
| Gender | Male vs. female | 1.40 (1.09 – 1.78) | 0.008 | 1.30 (0.91-1.85) | 0.2 | 1.23 (0.93 – 1.63) | 0.14 |  |  |
| Diabetes | Yes vs. no | 1.07 (0.76 – 1.51) | 0.7 |  |  | 0.98 (0.67 – 1.45) | 0.9 |  |  |
| Tobacco use | Yes vs. no | 0.92 (0.60 – 1.41) | 0.7 |  |  | 0.83 (0.51 – 1.36) | 0.5 |  |  |
| BMI | 1 kg/m^2^ increase | 0.99 (0.95 – 1.04) | 0.7 |  |  | 1.03 (0.98 – 1.09) | 0.2 |  |  |
| Bilirubin | 1 mg/dL increase | 1.09 (1.02 – 1.17) | 0.008 |  |  | 1.11 (1.04 – 1.19) | 0.003 |  |  |
| INR | 1-point increase | 4.46 (2.27 – 8.77) | <0.001 |  |  | 6.95 (3.39 – 14.26) | <0.001 |  |  |
| Albumin | 1 g/dL increase | 0.90 (0.88 – 0.93) | <0.001 |  |  | 0.90 (0.87 – 0.93) | <0.001 |  |  |
| Creatinine | 1 mg/dL increase | 4.70 (2.82 – 7.84) | <0.001 |  |  | 4.65 (2.65 – 8.13) | <0.001 |  |  |
| Platelet count | 10^3^/mm³ increase | 1.00 (1.00 – 1.00) | 0.7 |  |  | 1.00 (1.00 – 1.00) | 0.7 |  |  |
| Ascites | Yes vs. no | 1.60 (1.18 – 2.17) | 0.003 |  |  | 1.99 (1.41 – 2.82) | <0.001 |  |  |
| Encephalopathy | Yes vs. no | 2.15 (1.11 – 4.18) | 0.02 |  |  | 2.64 (1.40 – 4.99) | 0.003 |  |  |
| Esophageal or gastric varices | Yes vs. no | 1.39 (0.99 – 1.94) | 0.05 |  |  | 1.49 (1.00 – 2.19) | 0.05 |  |  |
| Child-Pugh score | 1-point increase | 1.12 (1.06 – 1.18) | <0.001 |  |  | 1.19 (1.13 – 1.26) | <0.001 |  |  |
| MELD score | 1-point increase | 1.10 (1.05 – 1.15) | <0.001 | 1.13 (1.07-1.23) | <0.001 | 1.13 (1.07 – 1.18) | <0.001 | 1.09 (1.06-1.13) | <0.001 |
| Abstinence | Yes vs. no | 0.48 (0.37 – 0.62) | <0.001 | 0.24 (0.11-0.52) | <0.001 | 0.41 (0.29 – 0.57) | <0.001 | 0.43 (0.34-0.57) | <0.001 |

Abbreviations: BMI, body mass index; CI, confidence interval; INR, international normalized ratio; MELD, model for end-stage liver disease

**Supplementary Table 3. Performance of the different prognostic models for 5-year liver-related death for the validation cohort comparing the MELD-Na or the MELD 3.0**

| **Models** | **AUC (95%CI)** | **p-Value** | **Brier score** | **p-Value** |
| --- | --- | --- | --- | --- |
| **Age, Child-Pugh score and abstinence at baseline** | 0.77 (0.68-0.85) | - | 0.166 | - |
| **MELD-Na** | 0.62 (0.50-0.74) | 0.05 | 0.206 | 0.03 |
| **MELD 3.0** | 0.63 (0.51-0.75) | 0.06 | 0.209 | 0.02 |

Abbreviations: AUC, area under the curve; CI, confidence interval; MELD, model for end-stage liver disease

**Supplementary Table 4: Performance of the different prognostic models for 5-year liver-related death with all available data**

|  | **Derivation dataset** | | | | | | **Validation dataset** | | | |
| --- | --- | --- | --- | --- | --- | --- | --- | --- | --- | --- |
| **Models** | **AUC (95%CI)** | **p-Value** | **Brier score** | **p-Value** | **AIC** | **BIC** | **AUC (95%CI)** | **p-Value** | **Brier score** | **p-Value** |
| **Age, Child-Pugh score and abstinence at baseline** | 0.73 (0.67-0.79) | - | 0.156 | - | 1066 | 1074 | 0.77 (0.69-0.86) | - | 0.167 | - |
| **Child-Pugh score at baseline** | 0.65 (0.58-0.71) | 0.007 | 0.174 | 0.04 | 1097 | 1100 | 0.66 (0.56-0.75) | 0.007 | 0.196 | 0.04 |
| **MELD score at baseline** | 0.68 (0.60-0.74) | 0.1 | 0.164 | 0.2 | 1089 | 1092 | 0.67 (0.56-0.77) | 0.047 | 0.186 | 0.08 |
| **Age, MELD score and abstinence at baseline** | 0.75 (0.68-0.81) | 0.1 | 0.150 | 0.7 | 1057 | 1065 | 0.75 (0.66-0.83) | 0.4 | 0.163 | 0.6 |

Abbreviations: AIC, Akaike’s information criterion; AUC, area under the curve; BIC, Bayesian information criterion; CI, confidence interval; MELD, model for end-stage liver disease

Note: Brier, AIC, BIC scores provide a global evaluation of the model: a lower Brier score, a lower AIC score and a lower BIC score indicate better performance.

**Supplementary Table 5: Performance of the different prognostic models for 5-year overall death**

|  | **Derivation dataset** | | | | | | **Validation dataset** | | | |
| --- | --- | --- | --- | --- | --- | --- | --- | --- | --- | --- |
| **Models** | **AUC (95%CI)** | **p-Value** | **Brier score** | **p-Value** | **AIC** | **BIC** | **AUC (95%CI)** | **p-Value** | **Brier score** | **p-Value** |
| **Age, Child-Pugh score and abstinence at baseline** | 0.69 (0.63-0.75) | - | 0.192 | - | 2949 | 2960 | 0.74 (0.65-0.83) | - | 0.200 | - |
| **Child-Pugh score at baseline** | 0.62 (0.54-0.67) | 0.01 | 0.208 | 0.01 | 3022 | 3026 | 0.63 (0.53-0.72) | 0.009 | 0.240 | 0.006 |
| **MELD score at baseline** | 0.64 (0.58-0.70) | 0.2 | 0.204 | 0.05 | 3015 | 3018 | 0.62 (0.52-0.71) | 0.01 | 0.235 | 0.02 |
| **Age, MELD score and abstinence at baseline** | 0.71 (0.65-0.77) | 0.1 | 0.187 | 0.7 | 2936 | 2947 | 0.72 (0.63-0.81) | 0.4 | 0.201 | 0.5 |

Abbreviations: AIC, Akaike’s information criterion; AUC, area under the curve; BIC, Bayesian information criterion; CI, confidence interval; MELD, model for end-stage liver disease

Note: Brier, AIC, BIC scores provide a global evaluation of the model: a lower Brier score, a lower AIC score and a lower BIC score indicate better performance.

**REFERENCES**

1. Thrift AP, Kanwal F, El-Serag HB. Prediction Models for Gastrointestinal and Liver Diseases: Too Many Developed, Too Few Validated. Clin Gastroenterol Hepatol 2016;14:1678-1680.

2. Steyerberg EW, Vickers AJ, Cook NR, Gerds T, Gonen M, Obuchowski N, Pencina MJ, et al. Assessing the performance of prediction models: a framework for traditional and novel measures. Epidemiology 2010;21:128-138.

3. Gerds TA, Cai T, Schumacher M. The performance of risk prediction models. Biom J 2008;50:457-479.

4. Hernandez-Orallo J FP, Ferri, C. Brier curves: a new cost-based visualisation of classifier performance. icml (available at <http://www.icml-2011.org/papers/366_icmlpaper.pdf>) 2011.

5. Reverter E, Tandon P, Augustin S, Turon F, Casu S, Bastiampillai R, Keough A, et al. A MELD-based model to determine risk of mortality among patients with acute variceal bleeding. Gastroenterology 2014;146:412-419 e413.

6. Rudler M, Bureau C, Carbonell N, Mathurin P, Saliba F, Mallat A, Massard J, et al. Recalibrated MELD and hepatic encephalopathy are prognostic factors in cirrhotic patients with acute variceal bleeding. Liver Int 2018;38:469-476.

7. Jepsen P, Watson H, Macdonald S, Vilstrup H, Jalan R. MELD remains the best predictor of mortality in outpatients with cirrhosis and severe ascites. Aliment Pharmacol Ther 2020.

8. Krzywinski M, Altman N. Points of significance: Nonparametric tests. Nat Methods 2014;11:467-468.

9. Altman DG, Vergouwe Y, Royston P, Moons KG. Prognosis and prognostic research: validating a prognostic model. BMJ 2009;338:b605.

10. Venkatraman ES. A permutation test to compare receiver operating characteristic curves. Biometrics 2000;56:1134-1138.

11. DeLong ER, DeLong DM, Clarke-Pearson DL. Comparing the areas under two or more correlated receiver operating characteristic curves: a nonparametric approach. Biometrics 1988;44:837-845.

12. Van Calster B, Nieboer D, Vergouwe Y, De Cock B, Pencina MJ, Steyerberg EW. A calibration hierarchy for risk models was defined: from utopia to empirical data. J Clin Epidemiol 2016;74:167-176.

13. Jr. HF. Regression modeling strategies. With applications to linear models, logistic regression, and survival analysis. New York: Springer-Verlag 2001.

14. Akaike H. Fitting autoregressive models for prediction. Ann Inst Stat Math 1969;21:243-247.

15. Schwartz G. Estimating the dimension of a model. Ann Stat 1978;6:461-464.
