# Supplementary figures and images for: A model for individualized prediction of liver-related death in outpatients with alcohol-associated cirrhosis
Source: Hepatol Commun. 2023 Aug 31;7(9):e0229. doi: 10.1097/HC9.0000000000000229 (PMC10476762; doi:10.1097/HC9.0000000000000229)

# Supplementary Fig 1. Flowchart of the validation dataset

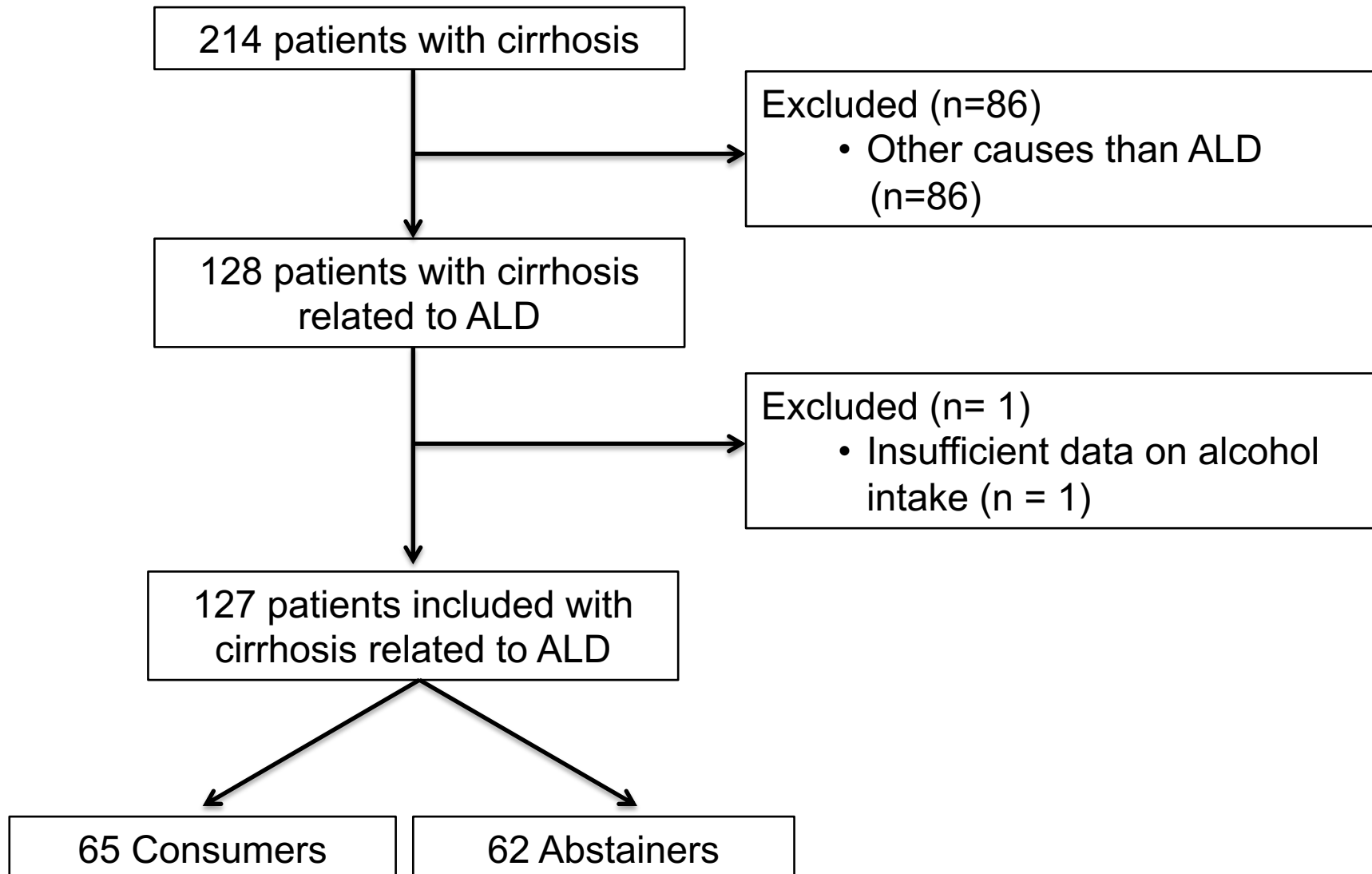

Supplement: Supplementary file 2 [file hc9-7-e0229-s002.pdf]

# Supplementary Fig 5. ROC curves of the different models in the validation dataset

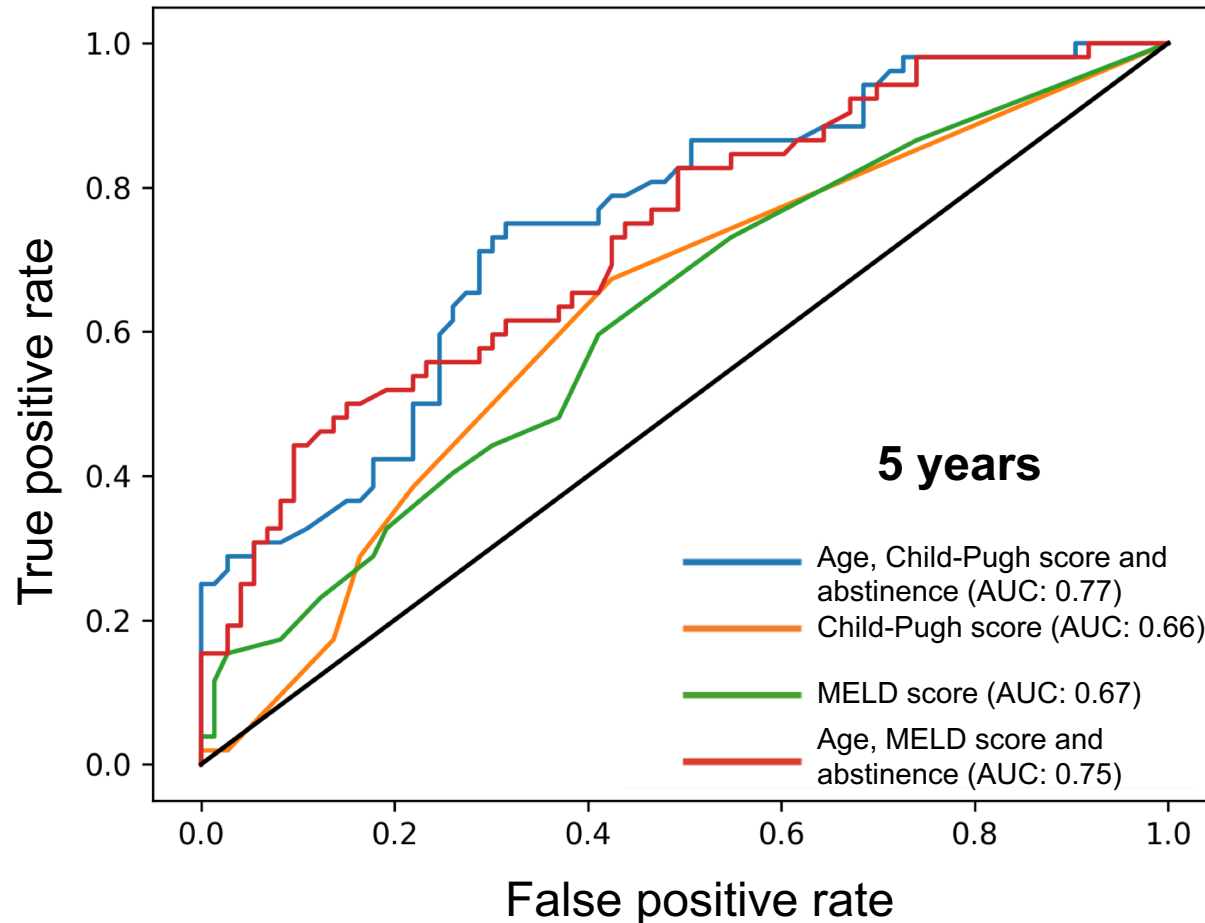

Supplement: Supplementary file 7 [file hc9-7-e0229-s007.pdf]
