# Supplementary material for: A model for individualized prediction of liver-related death in outpatients with alcohol-associated cirrhosis
Source: Hepatol Commun. 2023 Aug 31;7(9):e0229. doi: 10.1097/HC9.0000000000000229 (PMC10476762; doi:10.1097/HC9.0000000000000229)

Supplementary Fig 4. Charts predicting the risk of liver-related death at 5 years in patients who abstained from alcohol with the model including the MELD score instead of the Child-Pugh score

A.

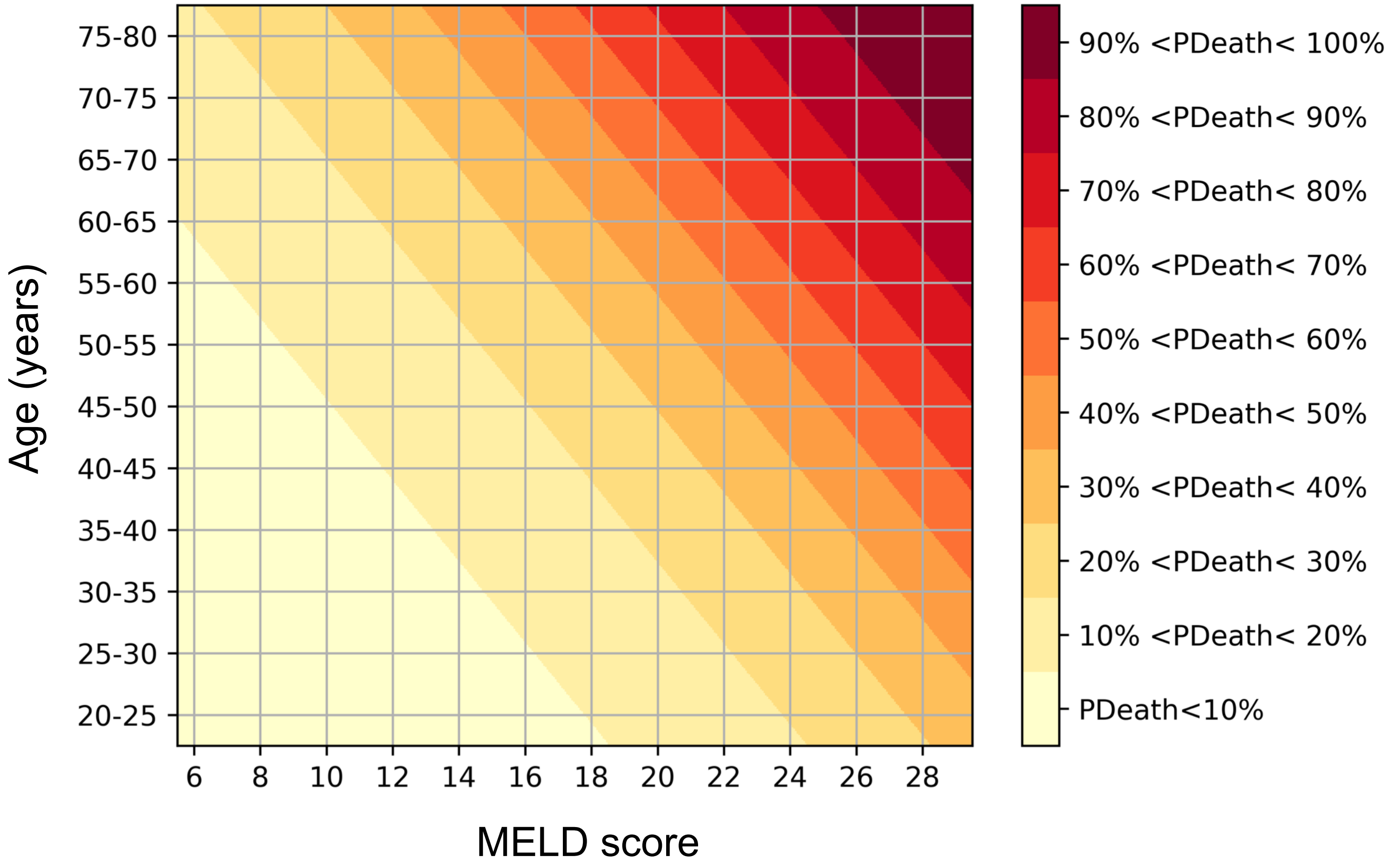

Supplement: Supplementary file 5 [file hc9-7-e0229-s005.pdf]
