# Supplementary material for: A model for individualized prediction of liver-related death in outpatients with alcohol-associated cirrhosis
Source: Hepatol Commun. 2023 Aug 31;7(9):e0229. doi: 10.1097/HC9.0000000000000229 (PMC10476762; doi:10.1097/HC9.0000000000000229)

Supplementary Fig 6. Calibration curves in the validation dataset

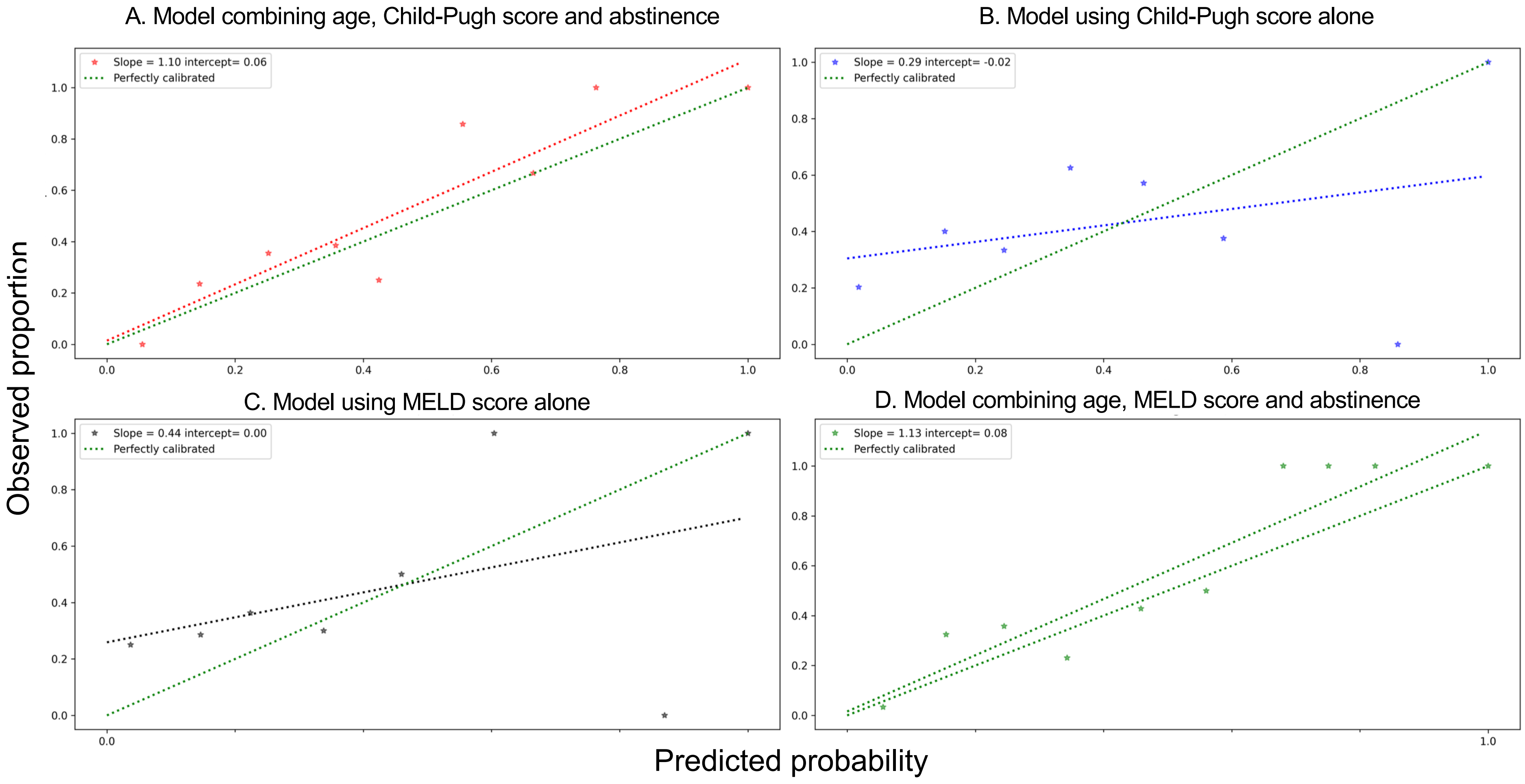

Supplement: Supplementary file 8 [file hc9-7-e0229-s008.pdf]
